# Comparison of the Effects of Interleukin-1 on Equine Articular Cartilage Explants and Cocultures of Osteochondral and Synovial Explants

**DOI:** 10.3389/fvets.2017.00152

**Published:** 2017-09-20

**Authors:** Christopher R. Byron, Richard A. Trahan

**Affiliations:** ^1^Department of Large Animal Clinical Sciences, Virginia–Maryland College of Veterinary Medicine, Virginia Tech, Blacksburg, VA, Unites States

**Keywords:** articular coculture, prostaglandin E2, bone alkaline phosphatase, matrix metalloproteinase-13, tumor necrosis factor-alpha, osteoarthritis, horse

## Abstract

Osteoarthritis (OA) is a ubiquitous disease affecting many horses. The disease causes chronic pain and decreased performance for patients and great cost to owners for diagnosis and treatment. The most common treatments include systemic non-steroidal anti-inflammatory drugs and intra-articular injection of corticosteroids. There is excellent support for the palliative pain relief these treatments provide; however, they do not arrest progression and may in some instances hasten advancement of disease. Orthobiologic treatments have been investigated as potential OA treatments that may not only ameliorate pain but also prevent or reverse pathologic articular tissue changes. Clinical protocols for intra-articular use of such treatments have not been optimized; the high cost of *in vivo* research and concerns over humane use of research animals may be preventing discovery. The objective of this study was to evaluate a novel *in vitro* articular coculture system for future use in OA treatment research. Concentrations and fold increases in various markers of inflammation (prostaglandin E_2_ and tumor necrosis factor-alpha), degradative enzyme activity [matrix metalloproteinase-13 (MMP-13)], cartilage and bone metabolism (bone alkaline phosphatase and dimethyl-methylene blue), and cell death (lactate dehydrogenase) were compared between IL-1-stimulated equine articular cartilage explant cultures and cocultures comprised of osteochondral and synovial explants (OCS). Results suggested that there are differences in responses of culture systems to inflammatory stimulation. In particular, the IL-1-induced fold changes in MMP-13 concentration were significantly different between OCS and cartilage explant culture systems after 96 h. These differences may be relevant to responses of joints to inflammation *in vivo* and could be important to the biological relevance of *in vitro* research findings.

## Introduction

Osteoarthritis (OA)-related joint pain affects a large proportion of the horse population resulting in chronic pain, decreased mobility, decreased performance, reduced quality of life, and high owner expense (1, 2). Common remedies for OA have included systemic administration of non-steroidal anti-inflammatory drugs (NSAIDs) and intra-articular injection of corticosteroids. However, these treatments are only palliative and do not modify the progression of OA. Furthermore, long-term NSAID use carries potentially serious side effects and corticosteroids may cause negative sequelae in articular cartilage (3). Therefore, orthobiologics (commonly termed regenerative therapies) have been used as potentially safer and more efficacious alternatives.

Orthobiologic techniques available for use in domestic animals include platelet-rich plasma, autologous conditioned serum [also known as IL-1 receptor antagonist protein (IL-1ra)], and autologous or allogeneic stem cells. Such treatments can improve function of equine joints (4–6). However, minimal beneficial effects may be found *in vitro* (7), and mechanisms of action remain unknown. In addition, clinical protocols for the use of orthobiologic treatments are currently not optimized. Therefore, there is a need for further research to refine clinical use of such therapies.

Although directly relevant to clinical application of treatments, use of live animal models is expensive, numbers of experimental subjects in studies may be insufficient to detect differences among groups (i.e., low statistical power), and there are welfare concerns over humane use of animals in research. The vast majority of rheumatology research in human and veterinary fields has been conducted with *in vitro* models including cells of only a single tissue type, cartilage. Cartilage damage has long been considered the hallmark of OA. However, molecular crosstalk between cartilage and subchondral bone cells is an important component of OA progression (8, 9). In addition, synoviocytes are important moderators of articular cartilage damage (10). *In vitro* models should account for this close relationship among articular tissues. There is a need for a physiologic *in vitro* model that can be used for the testing of potential OA treatments while reducing the use of live animals in research.

Coculture of articular tissues has been previously investigated, and results suggest that inclusion of multiple cell or tissue types changes molecular responses that may be more physiologic. Loss of glycosaminoglycans (GAGs) from cartilage, increase in expression of degradative enzymes, and decrease in expression of aggrecan in response to stimulation with IL-1 are partially abrogated by inclusion of synoviocytes in cartilage explant cultures (11). Coculture of bovine cartilage and subchondral bone improves chondrocyte survival compared with culture of cartilage alone (12). Coculture of canine articular cartilage and synovium seems to mimic responses of normal and osteoarthritic joints to stimuli (13, 14). Bovine chondrocyte expression patterns are altered when cartilage explants are cocultured with synovial explants (15). The cytokine profile of cocultured human cartilage and synovial explants obtained from patients with OA more closely represents the *in vivo* profile of osteoarthritic joints than monoculture of either tissue alone (16).

Despite the importance of cartilage, synovium, and subchondral bone in OA and data indicating inclusion of multiple articular tissue types in cultures results in more physiologic responses, coculture of cartilage, subchondral bone, and synovium has not been evaluated. The purpose of this study was to compare IL-1-induced expression of select metabolic markers in cultures containing cartilage explants alone versus cultures containing osteochondral and synovial explants (OCS). We hypothesized that changes in expression would differ between culture types. Results are expected to be useful in development of an *in vitro* culture model that more closely mimics *in vivo* articular responses to inflammatory stimulation than culture of single articular tissues alone.

## Materials and Methods

### Samples

Articular tissue samples (synovium, osteochondral explants, and cartilage explants) were collected from femoropatellar joints of five horses without clinical or gross evidence of degenerative joint disease that died as a result of causes unrelated to this study. Tissues from horses with synovial effusion, history of lameness attributable to stifle joints, or with gross signs of degenerative joint disease (hyaline cartilage erosion, score lines, discoloration, or fibrillation) were not used (17). No experiments were performed on animals prior to euthanasia. Use of cadaver tissues was in accordance with an approved IACUC protocol (number 14-259).

### Collection of Samples and Articular Tissue Culture

Immediately after death or euthanasia (*via* IV injection of an overdose of pentobarbital), samples of synovium, osteochondral explants, and cartilage explants were aseptically collected from femoropatellar joints of horses. Synovial tissue samples without fibrous joint capsule were collected with a biopsy punch (diameter, 6 mm; Integra Miltex, Plainsboro NJ, USA) from the dorsolateral aspect of the joint. Then, osteochondral explants (diameter, 7.9 mm; cartilage depth, approximately 2 mm; subchondral bone depth, approximately 4 mm) were collected from the axial aspect of the lateral trochlear ridge with a coring reamer (TEKTON Hollow Punch, Michigan Industrial Tools, Grand Rapids, MI, USA). Cartilage explants without subchondral bone (diameter, 7.9 mm) were also collected with a coring reamer (TEKTON Hollow Punch, Michigan Industrial Tools, Grand Rapids, MI, USA) from the axial aspect of the lateral trochlear ridge. Tissue samples were incubated for 1 h at 25°C in physiologic saline (0.9% NaCl) solution containing 1% penicillin and streptomycin (Thermo Fisher Scientific, Waltham, MA USA). Then, articular tissues were transferred to 12-well coculture plates (Transwell, Corning Life Sciences, Tewksbury MA, USA; well diameter, 12 mm) with polyester membranes (thickness, 10 µm; pore size, 3 µm). For each OCS coculture well, two synovial tissue samples were placed in the bottoms of plate wells and one osteochondral explant was suspended in well inserts. The ratio of synovium to osteochondral explants was determined on the basis of articular synovium and cartilage surface area ratios in mammals (18). For cartilage only cultures, one cartilage explant was placed in each well without other articular tissues. Articular tissue samples were incubated at 37°C with 95% relative humidity and 5% carbon dioxide in Dulbecco’s Modified Eagle Medium containing 1% ascorbate-2-phosphate, 1% insulin–transferrin–selenium, 1% penicillin and streptomycin, and 50 μg/mL l-proline (2.8 mL of medium/well; Corning Life Sciences, Tewksbury MA, USA).

Each treatment group was cultured with duplicate samples. Tissues were allowed to equilibrate in culture for 48 h prior to initiation of treatments. Groups included cocultures (OCS) with and without IL-1 [10 ng/mL (11, 19); rhIL-1β, R&D Systems, Minneapolis, MN, USA] and cartilage explants with and without IL-1. Media were replenished and collected at 48 and 96 h. Samples were stored at −80°C until analysis. Sample storage times were 6–10 months and all samples were analyzed concurrently. Assays included prostaglandin E_2_ (PGE_2_), tumor necrosis factor-alpha (TNF-alpha), matrix metalloproteinase-13 (MMP-13), dimethyl-methylene blue (DMMB), bone alkaline phosphatase (BAP), and lactate dehydrogenase (LDH).

### PGE_2_ Assay

The concentration of PGE_2_ in spent media was determined by use of a commercial colorimetric assay (R&D Systems, Minneapolis, MN, USA) following the directions of the manufacturers. Briefly, media (dilution, 1:25) were incubated in assay buffer containing primary anti-PGE_2_ antibody for 1 h at 25°C. Then, 50 µL of horseradish peroxidase-conjuated PGE_2_ solution were added to each well and incubated for 2 h at 25°C. Assay wells were washed four times, and 200 µL of a solution containing hydrogen peroxide and tetramethylbenzidine were added to each well. Plates were incubated for 30 min at 25°C. A stop solution (100 µL) of 2 N sulfuric acid was added to each well. Absorbance was measured at 450 nm (Molecular Devices SpectraMax M5, Sunnyvale, CA, USA) and PGE_2_ concentrations determined by comparison to a standard curve with 4-parameter logistic regression.

### TNF-Alpha Assay

The concentration of TNF-alpha in media was determined with a commercial assay (Thermo Scientific, Waltham, MA, USA) in accordance with the manufacturer’s instructions. Briefly, plate wells were coated with anti-TNF-alpha antibody and 100 µL of media (dilution, 1:2) were added to each well. Plates were incubated for 1 h at 25°C and then washed three times. Anti-equine TNF-alpha detection antibody was added to each well (100 µL/well) and plates were incubated for 1 h at 25°C. Wells were washed three times and 100 µL of a Streptavidin-horseradish peroxidase solution were added to each well. Plates were incubated for 30 min at 25°C. Wells were washed three times, 100 µL of a substrate solution were added to each well, and plates were incubated for 20 min in the dark at 25°C. The reaction was stopped by the addition of 100 µL of a 0.16 M sulfuric acid to each well. Optical density was measured at 450 nm (Molecular Devices SpectraMax M5, Sunnyvale, CA, USA) and TNF-alpha concentrations were determined by comparison with a standard curve.

### MMP-13 Assay

Stored media were assayed to detect MMP-13 with a commercially available kit (RayBiotech, Norcross, GA, USA) in accordance with the instructions of the manufacturer. Briefly, 100 µL of prepared standard and test media was incubated at 25°C for 2.5 h in assay wells coated with anti-MMP-13 antibody. Wells were washed four times with the supplied buffer and incubated at 25°C for 1 h with 100 µL of biotinylated anti-MMP-13 antibody. Wells were washed four times and incubated at 25°C for 45 min with 100 µL of Streptavidin solution. After washing four times, plated were incubated for 30 min at 25°C with 100 µL of 3,3,5,5′-tetramethylbenzidine solution and then the reaction was stopped by the addition of 0.2 M sulfuric acid. Optical density was measured immediately at 450 nm (Molecular Devices SpectraMax M5, Sunnyvale, CA, USA) and MMP-13 concentrations were determined *via* comparison with a standard curve and 4-parameter logistic regression.

### DMMB Assay

Media were digested in papain (0.5 mg/mL; Sigma-Aldrich, St. Louis, MO, USA) at 65°C for 4 h. The 1,9-dimethylmethylene blue assay (Sigma-Aldrich, St. Louis, MO, USA) was performed on digested media (dilution, 1:4) by use of the direct spectrophotometric method to measure the total GAG content in the spent media (20). Optical density was measured at 525 nm (Molecular Devices SpectraMax M5, Sunnyvale, CA, USA). Results were compared with a chondroitin sulfate standard curve to determine GAG concentrations.

### BAP Assay

Media were assayed to determine BAP concentrations with a commercially available kit (Quidel, San Diego, CA, USA) in accordance with the manufacturer’s instructions. Briefly, 125 µL of supplied assay buffer and 20 µL of sample media (dilution, 1:2) were added to plate wells precoated with anti-BAP antibody and incubated for 3 h at 25 C. Wells were washed four times and 150 µL of a 2-amino-2-methyl-1-propanol substrate solution were added to each well. Plates were incubated for 30 min at 25°C. The reaction was stopped by the addition of 100 µL of 0.5 N NaOH and optical density determined with a plate reader at 405 nm (Molecular Devices SpectraMax M5, Sunnyvale, CA, USA). Concentrations of BAP were determined *via* comparison with a standard curve generated with standard reagents supplied by the manufacturer.

### LDH Assay

Concentrations of LDH in media were determined with a commercially available assay (Roche, Basel, Switzerland). Briefly, 100 µL of sample media was incubated with 100 µL of reaction mixture containing diaphorase/NAD+, iodotetrazolium chloride, and sodium lactate in 96-well plates in the dark at 25°C for 30 min. Formazan was quantified as a measure of LDH activity by measuring absorbance at 492 nm on an automated microplate reader (Molecular Devices SpectraMax M5, Sunnyvale, CA, USA). Concentrations of LDH were determined by 4-parameter logistic regression.

### Data Analysis

Normality was assessed with probability plots. Concentrations of biomarkers were compared between positive and negative conditions (i.e., with and without IL-1β, respectively) within each combination of culture type group (OCS and cartilage) and time point (48 versus 96 h) using Friedman’s chi-square with horse as a blocking factor (SAS/STAT, SAS Institute, Cary, NC, USA). A logarithmic (base e) transformation was applied to the fold changes before any downstream analyses. Effects of culture type and time on the log fold changes were assessed using mixed model analysis of variance. Where appropriate *P*-values were adjusted for multiple comparisons using Bonferroni’s procedure. The linear model specified culture group, time, and interaction between group and time as fixed effects. Denominator degrees of freedom for the fixed effects were approximated using the Kenward–Roger method. Horse identification was specified as the random effect. Within the specified interaction, the following comparisons were extracted: (1) time point 48 versus time point 96 for each group and (2) OCS versus cartilage at each time point. For all analysis of variance models, residuals were inspected to verify that the errors followed a normal distribution with constant variance. Values of *P* < 0.05 were considered significant.

## Results

### PGE_2_

**Figure 1:**
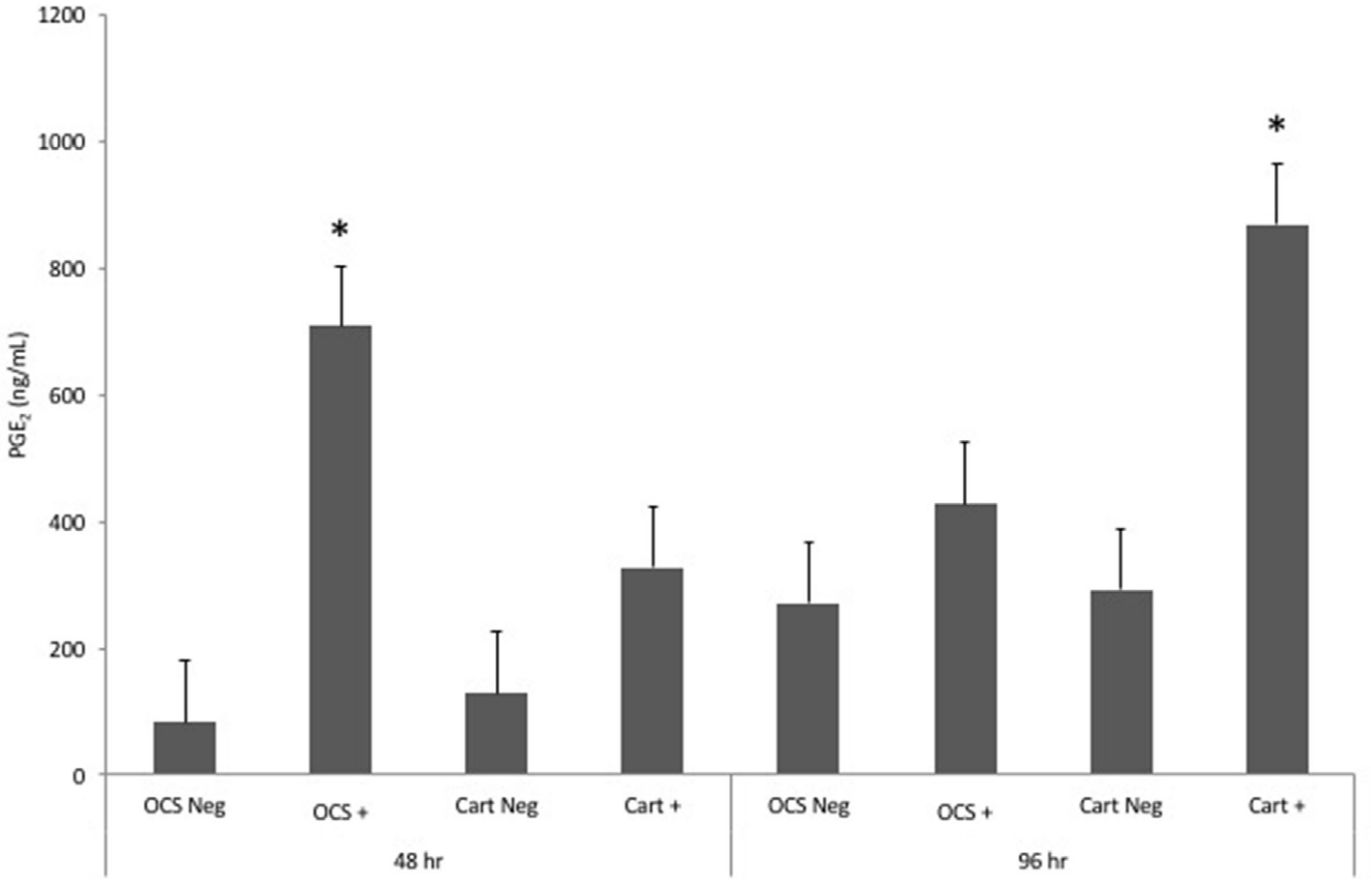
Mean ± SE concentrations of PGE_2_ in media samples of cultures containing osteochondral and synovial explants (OCS) or cartilage explants alone (Cart) that were unstimulated (Neg) or stimulated with IL-1 (10 ng/mL; +) at 48 and 96 h after initiation of treatments. *Within a culture type and time, concentration for IL-1-stimulated culture is significantly different from than that for the unstimulated culture.

Stimulation of OCS explant cultures with IL-1 resulted in a mean 8.4- and 1.6-fold increase in the media PGE2 concentration at 48 and 96 h, respectively (Figure 1). Stimulation of cartilage explant cultures with IL-1 resulted in a 2.6- and 3.0-fold increase in the PGE2 concentration at 48 and 96 h, respectively. The IL-1-stimulated OCS explant culture, PGE2 concentration was significantly (*P* = 0.03) higher than the concentration for unstimulated OCS explants at 48 h. The IL-1-stimulated cartilage explant culture PGE2 concentration was significantly (*P* = 0.03) higher than the concentration for unstimulated cartilage explants at 96 h. Differences between IL-1-stimulated and unstimulated culture PGE2 concentrations were not significantly different for cartilage at 48 h and OCS cultures at 96 h. Comparisons of fold changes in PGE2 concentrations between IL-1 stimulated and unstimulated explants were not significantly different between culture types at 48 and 96 h or between 48 and 96 h times for each culture type.

### Tumor Necrosis Factor-Alpha

**Figure 2:**
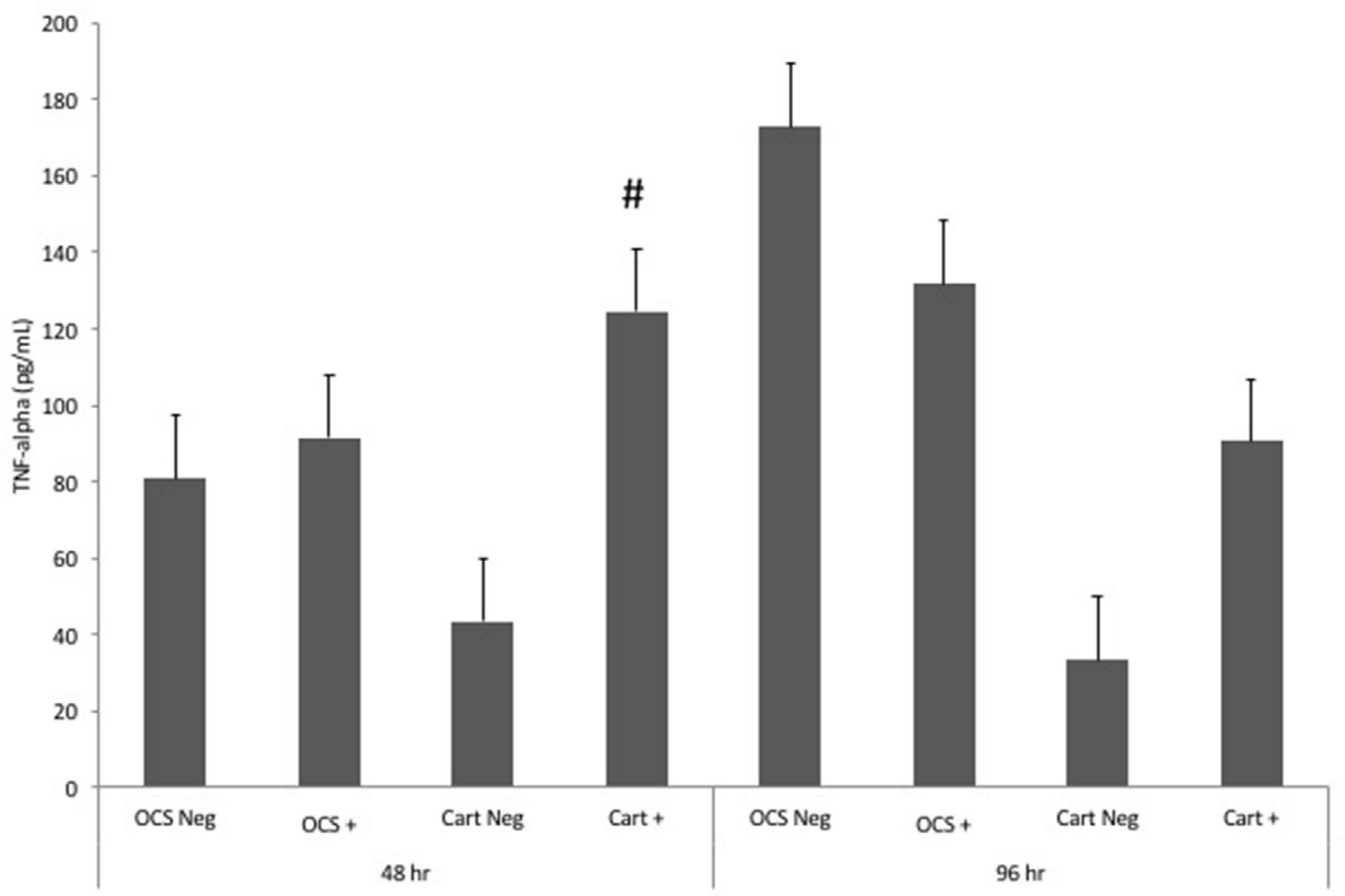
Mean ± SE concentrations of tumor necrosis factor-alpha (TNF-alpha) in media samples of cultures containing osteochondral and synovial explants (OCS) or cartilage explants alone (Cart) that were unstimulated (Neg) or stimulated with IL-1 (10 ng/mL; +) at 48 and 96 h after initiation of treatments. ^#^Within a time, the fold increase in TNF-alpha concentration between unstimulated and stimulated cultures is significantly different between culture types.

Stimulation of OCS explant cultures with IL-1 resulted in a mean 1.1-fold increase in the media TNF-alpha concentration at 48 h and a 1.3-fold decrease in TNF-alpha concentration at 96 h (Figure 2). Stimulation of cartilage explant cultures with IL-1 resulted in a 2.9- and 2.7-fold increase in the TNF-alpha concentration at 48 and 96 h, respectively. However, differences between IL-1-stimulated and unstimulated culture TNF-alpha concentrations were not significantly different for cartilage or OCS explant cultures at 48 or 96 h. At 48 h, the fold increase in TNF-alpha concentration between IL-1-stimulated and unstimulated cultures was significantly (*P* = 0.04) greater for cartilage versus OCS cultures. Comparisons of fold changes in TNF-alpha concentrations between IL-1 stimulated and unstimulated explants were not significantly different between culture types at 96 h or between 48 and 96 h times for each culture type.

### Matrix Metalloproteinase-13

**Figure 3:**
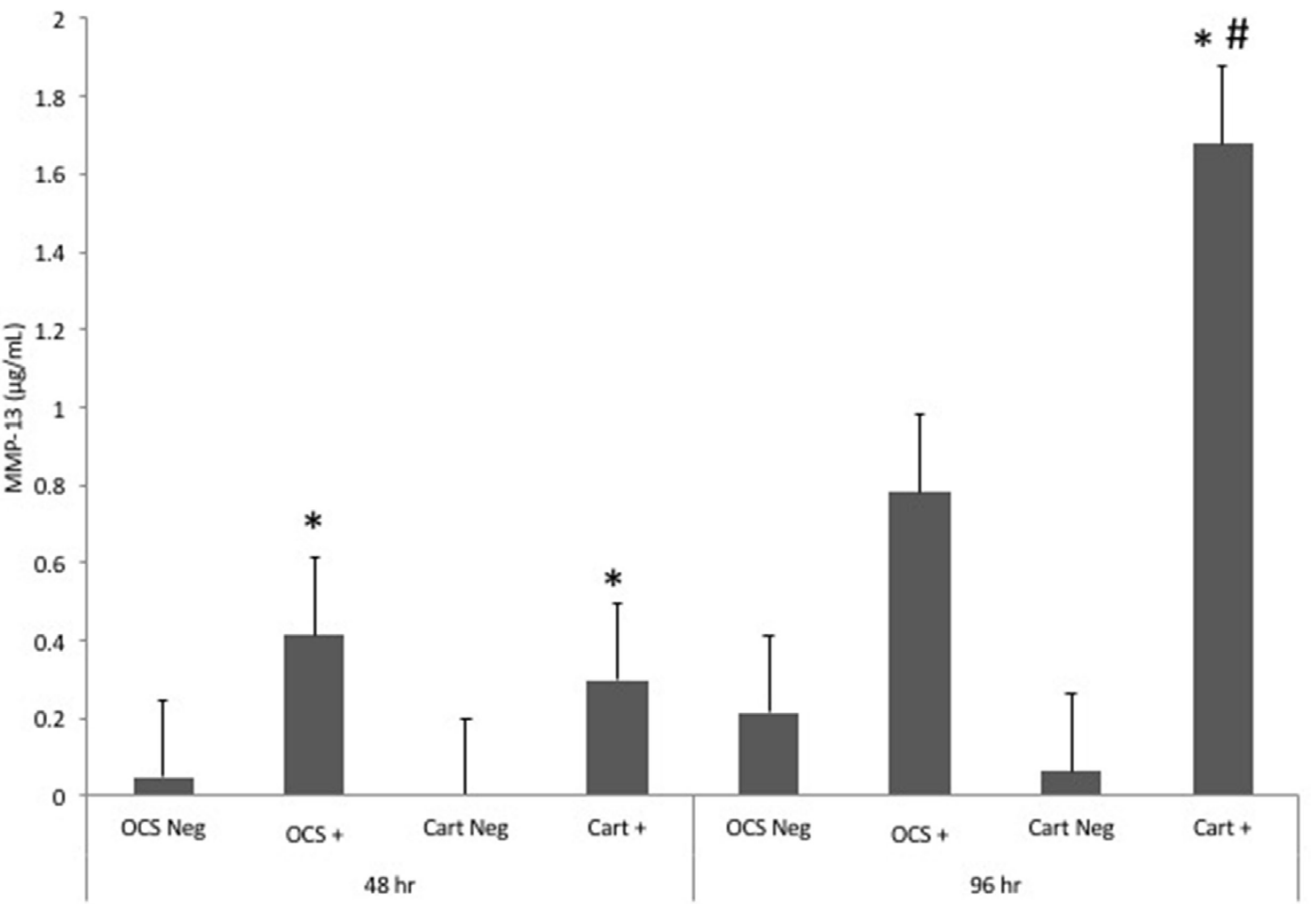
Mean ± SE concentrations of matrix metalloproteinase-13 (MMP-13) in media samples of cultures containing osteochondral and synovial explants (OCS) or cartilage explants alone (Cart) that were unstimulated (Neg) or stimulated with IL-1 (10 ng/mL; +) at 48 and 96 h after initiation of treatments.

Stimulation of OCS explant cultures with IL-1 resulted in a mean 8.4- and 3.6-fold increase in the media MMP-13 concentration at 48 and 96 h, respectively (Figure 3). Stimulation of cartilage explant cultures with IL-1 resulted in a 74- and 26-fold increase in the MMP-13 concentration at 48 and 96 h, respectively. The IL-1-stimulated OCS explant culture MMP-13 concentration was significantly (*P* = 0.03) higher than the concentration for unstimulated OCS explants at 48 h but was not significantly different at 96 h. The IL-1-stimulated cartilage explant culture MMP-13 concentration was significantly (*P* = 0.03) higher than the concentration for unstimulated cartilage explants at 48 and 96 h. At 96 h, the fold increase in MMP-13 concentration between IL-1-stimulated and unstimulated cultures was significantly (*P* = 0.02) greater for cartilage versus OCS cultures. Comparisons of fold changes in MMP-13 concentrations between IL-1 stimulated and unstimulated explants were not significantly different between culture types at 48 h or between 48 and 96 h times for each culture type.

### Dimethyl-Methylene Blue

**Figure 4:**
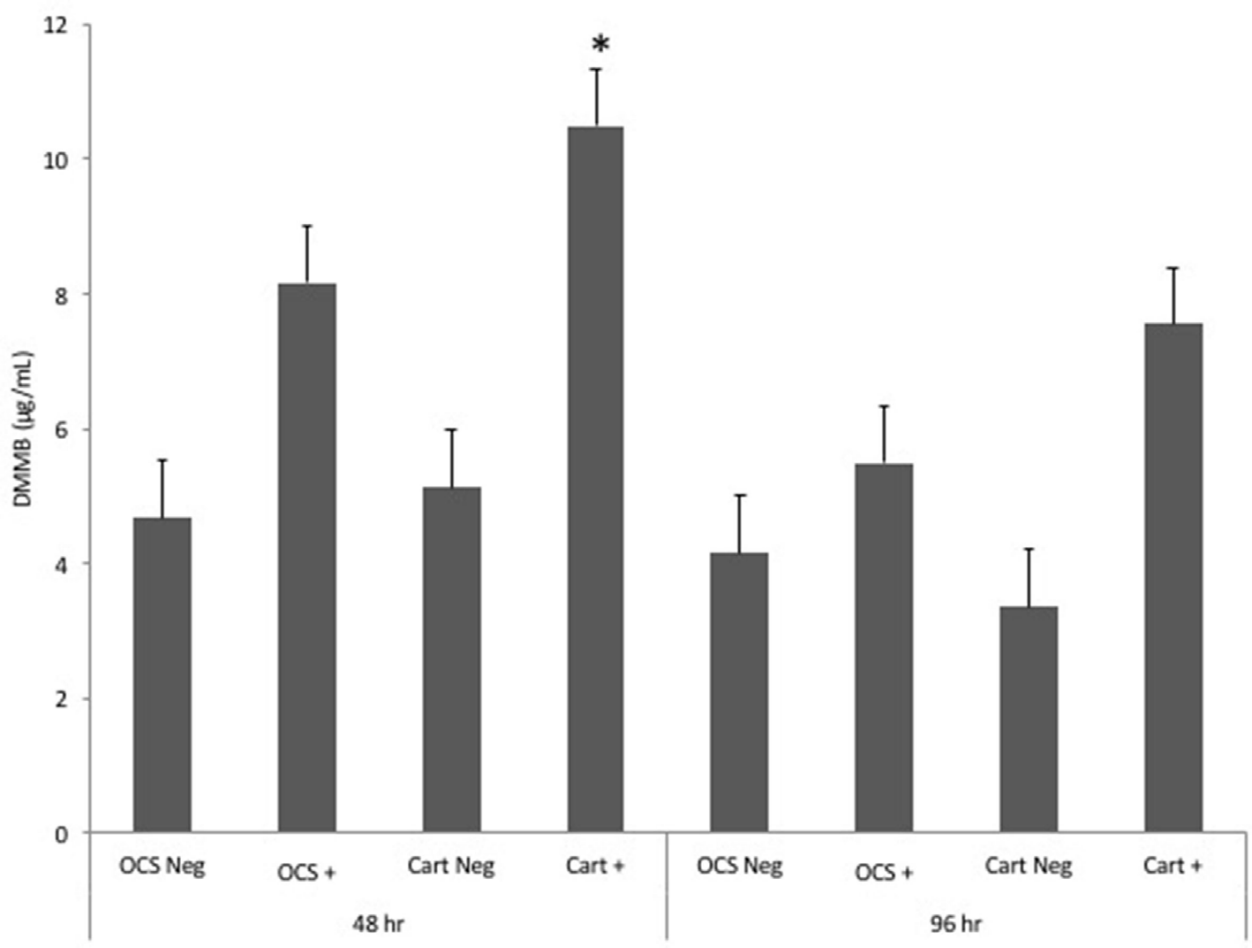
Mean ± SE concentrations of dimethyl-methylene blue (DMMB) in media samples of cultures containing osteochondral and synovial explants (OCS) or cartilage explants alone (Cart) that were unstimulated (Neg) or stimulated with IL-1 (10 ng/mL; +) at 48 and 96 h after initiation of treatments.

Stimulation of OCS explant cultures with IL-1 resulted in a mean 1.7- and 1.3-fold increase in the media GAG concentration at 48 and 96 h, respectively (Figure 4). Stimulation of cartilage explant cultures with IL-1 resulted in a 2.1- and 2.3-fold increase in the GAG concentration at 48 and 96 h, respectively. The IL-1-stimulated cartilage explant culture GAG concentration was significantly (*P* = 0.03) higher than the concentration for unstimulated cartilage explants at 48 h but was not significantly different for cartilage explants at 96 h or for OCS explants at either 48 or 96 h times. Comparisons of fold changes in GAG concentrations between IL-1-stimulated and -unstimulated explants were not significantly different between culture types at 48 or 96 h times or between 48 and 96 h times for each culture type.

### Bone Alkaline Phosphatase

**Figure 5:**
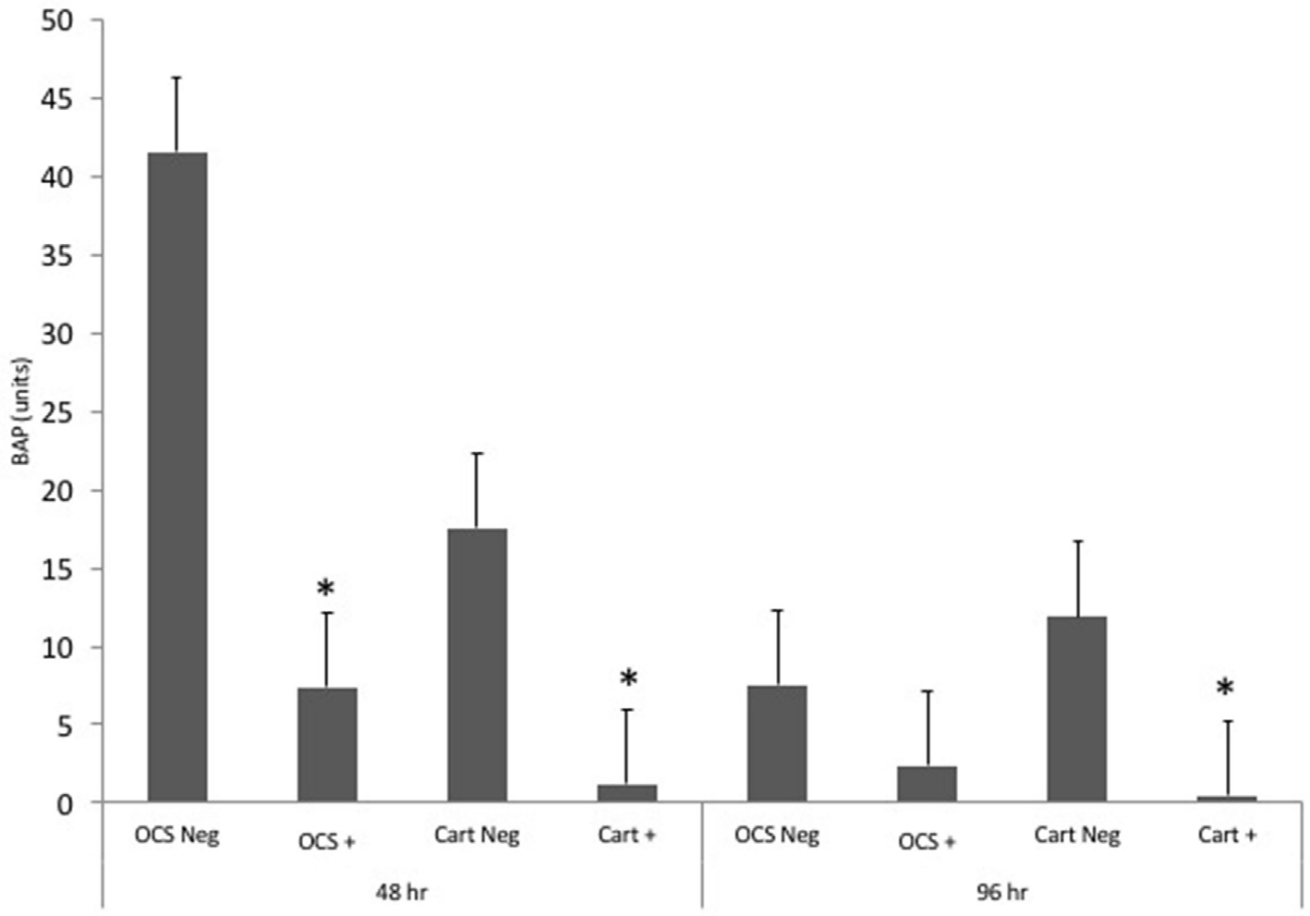
Mean ± SE concentrations of bone alkaline phosphatase (BAP) in media samples of cultures containing osteochondral and synovial explants (OCS) or cartilage explants alone (Cart) that were unstimulated (Neg) or stimulated with IL-1 (10 ng/mL; +) at 48 and 96 h after initiation of treatments.

Stimulation of OCS explant cultures with IL-1 resulted in a mean 5.6- and 3.2-fold decrease in the media BAP concentration at 48 and 96 h, respectively (Figure 5). Stimulation of cartilage explant cultures with IL-1 resulted in a 14.1- and 24.3-fold decrease in the BAP concentration at 48 and 96 h, respectively. The IL-1-stimulated cartilage explant culture BAP concentration was significantly lower than the concentration for unstimulated cartilage explants at 48 and 96 h (*P* = 0.03 and 0.04, respectively). The IL-1-stimulated OCS explant BAP concentration was significantly (*P* = 0.03) lower than the concentration for unstimulated cartilage explants at 48 h but was not significantly different at 96 h. Comparisons of fold changes in BAP concentrations between IL-1 stimulated and unstimulated explants were not significantly different between culture types at 48 and 96 h, although the values of *P* were nearly significant (*P* = 0.055 and 0.051, respectively). Comparisons of fold changes were not significant between 48 and 96 h times for each culture type.

### Lactate Dehydrogenase

**Figure 6:**
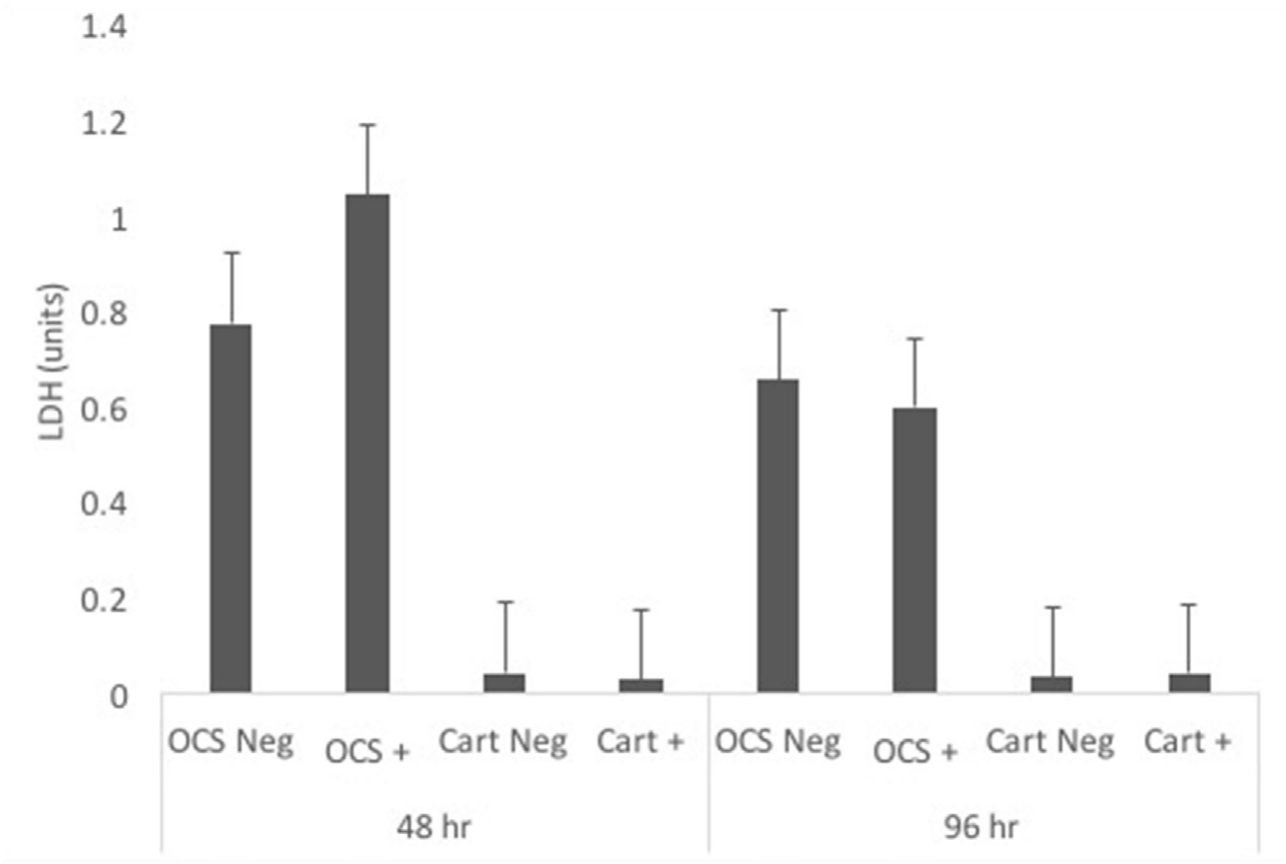
Mean ± SE concentrations of lactate dehydrogenase (LDH) in media samples of cultures containing osteochondral and synovial explants (OCS) or cartilage explants alone (Cart) that were unstimulated (Neg) or stimulated with IL-1 (10 ng/mL; +) at 48 and 96 h after initiation of treatments.

Concentrations of LDH were not significantly different between IL-1-stimulated and unstimulated explants for either culture type at 48 or 96 h (Figure 6). Likewise, no significant differences in fold change comparisons were found.

## Discussion

This study was conducted to compare responses of various cell and tissue metabolic markers to IL-1 stimulation in monoculture (cartilage explants only) and coculture (OCS) systems. These included markers of inflammation (PGE_2_ and TNF-alpha), extracellular matrix degradation (MMP-13 and DMMB assays), bone metabolism (BAP), and cell viability (LDH). Results suggested that there are differences in responses of culture systems to inflammatory stimulation. In particular, the IL-1-induced fold changes in MMP-13 concentration were significantly and substantially different between OCS and cartilage explant culture systems. These differences may be relevant to responses of joints to inflammation *in vivo* and could be important to the biological relevance of *in vitro* research findings.

In response to IL-1 stimulation, both OCS and cartilage explant cultures had an increase in PGE_2_ concentration. The increase was greatest and statistically significant at 48 h for OCS cultures and at 96 h for cartilage explant cultures. This finding may indicate temporal differences in PGE_2_ responses for these culture systems. However, the magnitude of the increase in PGE_2_ concentration was not significantly different between culture types at 48 or 96 h. Also, the magnitude of the increase in PGE_2_ concentration was similar at each time point for OCS and cartilage cultures. These findings suggest that, while there may be temporal differences in PGE_2_ expression between cartilage explant monocultures and articular tissue cocultures, the responses are overall similar. To our knowledge, no other studies have compared the PGE_2_ expression responses between cartilage explant cultures and articular tissue cocultures. Although an explanation for temporal differences in PGE_2_ expression between culture types is not known, we believe it is due to enhanced expression of anti-inflammatory cytokines in OCS cultures. Other investigators have shown that synovial tissue produces IL-1ra, but cartilage explants do not (16). This would lead to reduction in IL-1 response in cocultures over time, which is consistent with our finding of lower PGE_2_ expression at 96 h in the OCS group.

Stimulation of cartilage explants with IL-1 resulted in a significant increase in TNF-alpha expression at 48 h, whereas stimulation of OCS explants did not result in a significant change in expression at either time point evaluated. Although the response of both culture systems was modest, there was a significant difference in the magnitude of the IL-1-induced increase in TNF-alpha expression between cartilage and OCS explant cultures at 48 h. The modest increase in expression of TNF-alpha in these culture systems is not unexpected. Human cartilage and synovial tissue obtained from osteoarthritic joints have low expression of TNF-alpha when culture alone or together in a coculture system (16). Other authors found that synovial fluid concentrations of TNF-alpha do not increase in joints with various types of damage (21) or in carpal joints with pathologic changes related to OA (22). However, findings of another study (23) indicate TNF-alpha concentrations increase in joints with osteochondrosis dissecans or acute trauma. On the basis of these results, it seems that the TNF-alpha response to inflammation and joint damage is variable. Our results indicated a mild decrease in TNF-alpha for OCS cultures at 96 h; this result was not significant and the difference is likely attributable to variability in response among horses and modest protein expression. The differences in findings may be attributable to characteristics of inflammation and trauma or to the articular tissues (cartilage, synovium, or subchondral bone) involved. Further research is warranted to determine the contributions of each tissue type to articular expression of TNF-alpha.

Of the biomarkers evaluated in this study, the response of MMP-13 expression to IL-1 stimulation was the greatest in both types of cultures. Both cartilage and OCS explant cultures substantially increased MMP-13 expression in response to IL-1. In particular, cartilage explant cultures exposed to IL-1 had very high expression of MMP-13 protein. The magnitude of the MMP-13 response to IL-1 was significantly greater for cartilage explants compared with OCS explants at 96 h. This finding indicates a substantial difference between these culture systems in the inflammation-induced expression of MMP-13. The inclusion of synovium and subchondral bone in culture seemed to partially abrogate the increase in MMP-13. Although we did not determine the individual contributions of synovium and subchondral bone to this result, this difference in response seems to be biologically relevant. Other authors (15) found that coculture of cartilage with synovial tissue alters expression of MMP-13. In another study (11), responses of cartilage explants were compared with those of cartilage and synovium cocultures; results indicated no significant differences between these groups in expression of MMP-13 mRNA after 96 h of exposure to IL-1. In contrast to our results, other authors reported that general matrix metalloproteinase activity is enhanced by coculture of synovium with cartilage explants (16). Inclusion of subchondral bone in the OCS group of our study may have downregulated MMP-13. This difference in results between the present study and that other study suggest that the tissue composition of *in vitro* culture systems can have a large effect on expression of MMP-13. Unfortunately, the design of our study does not allow differentiation of the effects of each individual tissue type. In light of this, further investigation seems warranted to determine similarities between of *in vitro* coculture systems and *in vivo* responses of joints.

Loss of extracellular matrix GAG into culture media indirectly indicates activities of certain degradative enzymes. Results of other studies indicate the effects of coculture on loss of cartilage GAG are variable. Coculture of equine cartilage and synovium protects against IL-1-induced loss of GAG from cartilage explants (11). However, coculture of human synovium with cartilage obtained from osteoarthritic joints results in a decrease in GAG production compared with monocultures of cartilage alone (16). Coculture of cartilage and synovium did not have a significant effect on release of GAG into culture media in either of those studies. Likewise, results of the present study did not indicate a significant effect of synovial and subchondral bone coculture with cartilage on IL-1-induced release of GAG into media. These findings suggest that coculture of osteoarthritic cartilage with other articular tissues has an effect on extracellular matrix GAG content, which is primarily attributable to changes in GAG production, but the effects on cultures in acute inflammatory conditions are variable.

Bone alkaline phosphatase has been used as a biomarker of bone turnover in humans and horses (24, 25). Exposure of rabbit chondrocytes to IL-1 dramatically decreases production of BAP (26). Interleukin-1 decreases bone formation in adult rats (27). The BAP expression of human osteoblasts decreases after exposure to IL-1 (28). Other authors found that IL-1 increases BAP expression (29). Although results of the present study did not indicate significant differences between culture types with regard to IL-1-induced changes in BAP expression, these results were very nearly significant. This suggests that inclusion of multiple articular tissue types in culture may have an effect on BAP expression, as would be expected considering molecular crosstalk between bone and cartilage is an important component of OA (30). The decrease in BAP expression after IL-1 exposure in this study was somewhat unexpected, considering synovial fluid levels in horses increase after joint injury. Other authors found that synovial fluid concentrations of BAP are higher in equine carpal joints with osteochondral injury than in normal carpal joints (24); however, metacarpophalangeal joints with and without injury did not significantly differ in that study. Results of another study of racehorses differed (31); BAP concentrations in fetlock joints of Thoroughbred racehorses with injury were significantly higher than in uninjured joints. Likewise, other authors have found signficantly higher BAP concentrations in carpal and fetlock joints of horses with cartilage damage compared with contralateral joints (32). We used articular tissues obtained from femoropatellar joints of horses. There are differences in BAP expression among joints (24). Prior studies evaluating equine articular BAP concentrations have primarily evaluated distal joints. Expression of BAP in the femoropatellar joint may differ from other joints because of differences in anatomic location and biomechanical forces (primarily shear rather than compression).

No significant differences were detected in LDH concentrations between untimulated and IL-1 stimulated cultures or in fold changes between culture types at 48 or 96 h. This finding indicates minimal cytotoxicity in cartilage explant and OCS cocultures. These results were similar to results of another study in which human OA cartilage was cultured with or without synovium (16); minimal cytotoxicity in cultures up to 21 days was detected *via* LDH release in that study. In another study, coculture of bovine synovial fibroblasts with chondrocytes protected against cell membrane damage secondary reactive oxygen species exposure (33).

Both subchondral bone (9) and synovial (10) cells are important in the progression of OA. In addition, molecular crosstalk between cartilage and subchondral bone is an important contributor to the pathogenesis of OA (30). Accordingly, the coculture system investigated in this study was intended to account for physiologic responses of all major articular tissues. In contrast, traditional *in vitro* models of joint disease only include chondrocytes or cartilage explants; results of such studies may not be directly applicable to joints in living animals. Other authors have investigated use of engineered articular cocultures comprised of osteogenic and chondrogenic mesenchymal stem cells (34) or chondrocytes and macrophages (35) in scaffolds to mimic *in vivo* responses. While these approaches may account for interactions among articular cells, they require additional processing of tissues and do not replicate native interactions between cells and the extracellular matrix.

This study had several limitations. The low (*n* = 5) number of horses included may have precluded detection of small differences among groups. In addition, horses of various ages and breeds were included, which may have contributed to high variability in responses among tissues from these animals. Responses of tissues to inflammatory stimulation was only investigated at 48 and 96 h times. There may be temporal differences in molecular responses that were not detected at these time points. Also, other investigators have maintained articular cocultures for substantially longer times (21 days) (16), which may be more relevant to long-term *in vivo* joint tissue responses. Another potential limitation is the use of IL-1 for induction of an inflammation to mimic an articular OA environment. Naturally occurring OA involves upregulation of multiple inflammatory cytokines. However, IL-1 known to be a major component of the inflammatory response in osteoarthritic joints of horses and is a well-established method for *in vitro* joint disease testing (36–38).

This study was conducted to compare responses of a novel *in vitro* articular coculture system with that of another *in vitro* model of joint physiology (cartilage explant monoculture). Results indicated overall similarity in outcomes. However, there were some notable differences that are likely attributable to molecular interplay between tissue types. Future OA research may benefit from the use of coculture systems, and findings may be more relevant to *in vivo* physiology. However, further research is needed to compare *in vitro* molecular responses with those of joints in horses. Validation of *in vitro* coculture systems would be valuable for testing of orthobiologic and other treatments prior to application in living animals with OA.

## Author Contributions

CB conceived of the study design, conducted experiments, analyzed data, and wrote and revised the manuscript. RT conducted experiments, analyzed data, and revised the manuscript.

## Conflict of Interest Statement

The authors declare that the research was conducted in the absence of any commercial or financial relationships that could be construed as a potential conflict of interest.
